# Effect of *Qianghuo Erhuang* Decoction on T Regulatory and T Helper 17 Cells in Treatment of Adjuvant-induced Arthritis in Rats

**DOI:** 10.1038/s41598-017-17566-w

**Published:** 2017-12-08

**Authors:** Can Qian, Mei Kuang, Yong Wang

**Affiliations:** 1 Department of Traditional Chinese Medicine, Southwest Hospital, Third Military Medical University, Chongqing, 400038 China; 2 Department of Phamarcology, Faculty of Pharmacy, Third Military Medical University, Chongqing, 400038 China

## Abstract

*QianghuoErhuang* Decoction (QED) is an effective recipe in treating rheumatoid arthritis. The present study aimed to explore the effects of QED on Treg and Th17 in adjuvant arthritis (AA) model. The study included 6 group rats: normal control group, AA group, AA + methotrexate (MTX) group, AA + high, moderate, and low dose QED groups. The arthritis score was significantly decreased in the MTX and high-dose QED groups compared with the AA group on days 24 and 28 (*P* < 0.01), respectively. The synovial tissue inflammation was attenuated by histological observation, and the proliferation of splenocytes was significantly inhibited in MTX and high-dose QED groups (*P* < 0.01). High-dose QED can up-regulated the percentage of Treg cells (*P* < 0.01) and down-regulated the percentage of Th17 cells (*P* < 0.05). Notably, the serum levels of IL-6, IL-17 and TNF-α were significantly decreased, while TGF-β levels were apparently elevated compared with AA group (*P* < 0.05, *P* < 0.01). Interestingly, moderate and low-dose QED had no such similar effects. In summary, high-dose QED had a therapeutic effect against adjuvant arthritis and regulated the related cytokine levels in serum. The underlying mechanism might be mediated via restoration of the imbalance in CD4^+^ T lymphocyte subsets, Treg/Th17.

## Introduction

Rheumatoid arthritis (RA) is a chronic autoimmune disease characterized by joint swelling, synovial inflammation and cartilage destruction^1^. The precise pathogenesis of RA has not been completely elucidated. However, it was reported that infiltration of a large number of CD4^+^ T lymphocytes and hyperplasia of synoviocytes in the synovial membrane had triggered and exacerbated synovitis in RA. Furthermore, the imbalance in T regulatory (Treg) and T helper 17 (Th17) cells was a crucial event in the pathogenesis of RA^2,3^.

Currently, non-steroidal anti-inflammatory drugs (NSAIDs), disease-modifying anti-rheumatic drugs (DMARDs), glucocorticoids and biologic drugs are usually recommended for RA treatment. However, these drugs are associated with varying degrees of side effects including gastrointestinal disorders, immunodeficiency, infection and humoral disturbances^4–6^, strongly suggesting the need to explore new therapeutic agents with low toxicity and high efficacy. Complementary therapies based on Chinese medicines can be recommended as an attractive alternative.

In traditional Chinese medicine (TCM), RA is categorized under arthromyodynia (BI syndrome in Mandarin) triggered by wind, cold, and dampness, as described in the Yellow Emperor’s Classic of Internal Medicine. Chinese clinicians have demonstrated damp-heat impeding syndrome and cold-damp impeding syndrome as the most common RA patterns in patients. Modern medicine demonstrated that most patients with RA during the active phase represent damp-heat impeding syndrome^7,8^. *Qianghuo Erhuang Decoction (QED)*, derived from ancient recipe *Qianghuo Shengshi Decoction* of Piwei Lun (Treatise on Spleen and Stomach) written by Li Dong Yuan, is composed of Qianghuo (Notopterygium incisum), Duhuo (Heracleum Linnaeus L.), Qinjiao (Gentiana macrophylla pall.), Yi Yiren (Coix lacryma-jobi L.), Ren Dongteng (Lonicerae japonica Thunb), Huangqin (Scutellaria baicalensis Georgi), Jianghuang (Curcuma longa L.), Ezhu (Curcuma zadoaria (Christm) Rosc.), Chuanxiong (Ligusticum chuanxiong Hort.), Gancao (Glycyrrhiza uralensis Fisch), which were combined in a ratio of 20:15:20:30:30:20:10:15:15:5. Treatment with this new traditional drug formulation could eliminate dampness and heat, activate blood circulation and relieve numbness, and therefore, could be more effective against rheumatism and heat-induced numbness. Modern pharmaceutical studies indicated that the ingredients were compatible with each other, and played an anti-inflammatory, analgesic and immune-regulatory role^9–11^.

QED has been previously used to treat more than 200 cases of active RA in our hospital. The preliminary data showed that disease active scale (DAS) 28 (4), erythrocyte sedimentation rate (ESR) and C-reactive protein (CRP) were significantly decreased, without any significant side effects within two months of treatment. Therefore, we further investigated the mechanism of QED in adjuvant-induced arthritis (AA) via the Treg/Th17 cell ratio using a rat model.

## Results

### Effects of different QED dosages on arthritis score

AA was developed 14 d after rats were immunized with Complete Freund’s Adjuvant (CFA), and the success rate in rat models was 71%. Peak inflammation occurred after 21 d. The AA rats displayed poor foraging ability and slow movement, with lameness in hind limb and showed decreased over all activity. The hind limb showed initial disease onset, with redness, swelling, heat and other inflammatory manifestations. Ankle and knuckle joints were greatly affected with arthrocele, which was symmetrical among few rats.

**Figure 1:**
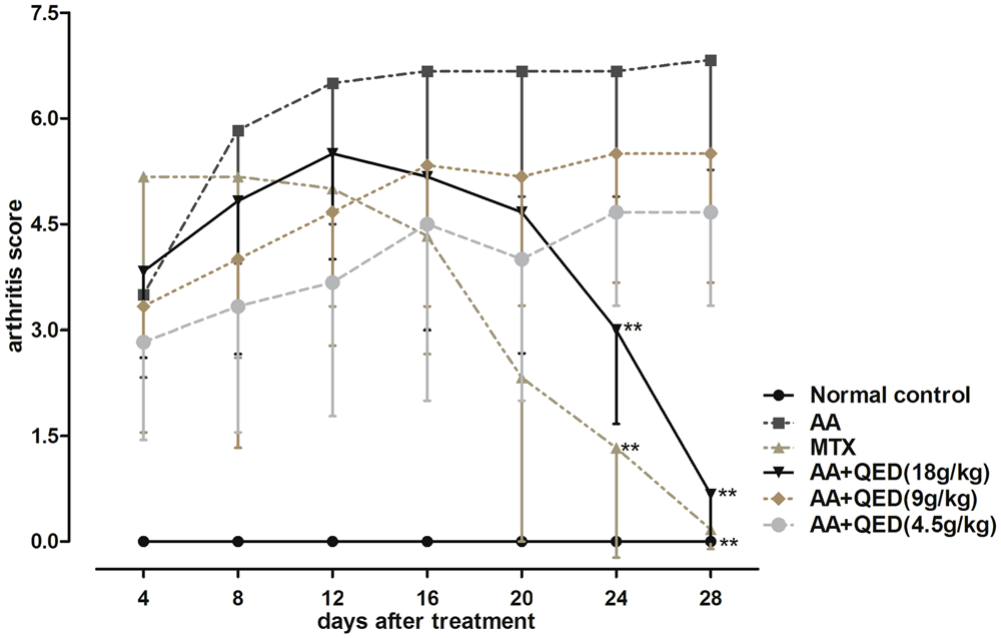
Effects of different QED dosages on arthritis score. Male SD rats were immunized with Mtb via subcutaneous injection at the base of the tail. AA rats were administered with saline intragastrically, MTX (0.4 mg/kg per week), high-, moderate-, and low-dose QED (daily dose: 18, 9, and 4.5 g/kg, respectively) (n = 6 for each group) for four weeks. Data were expressed as mean ± S.D (n = 6 each group). *P < 0.05, **P < 0.01, versus AA group.

The arthritis score estimated in rats reflected the degree of arthritis. On the 4^th^, 8^th^, 12^th^, 16^th^ and 20^th^ day after medication, scores were not statistically significant (*P* > 0.05) when each of the treatment group was compared with AA group. Scores of MTX and high-dose QED groups were obviously decreased on days 24 and 28 after medication, respectively. Score differences between the groups on these days showed statistical significance (*P* < 0.01), indicating decrease in arthritis symptoms (Fig. 1).

### Effects of different QED dosages on the thickness of hind paws

**Figure 2:**
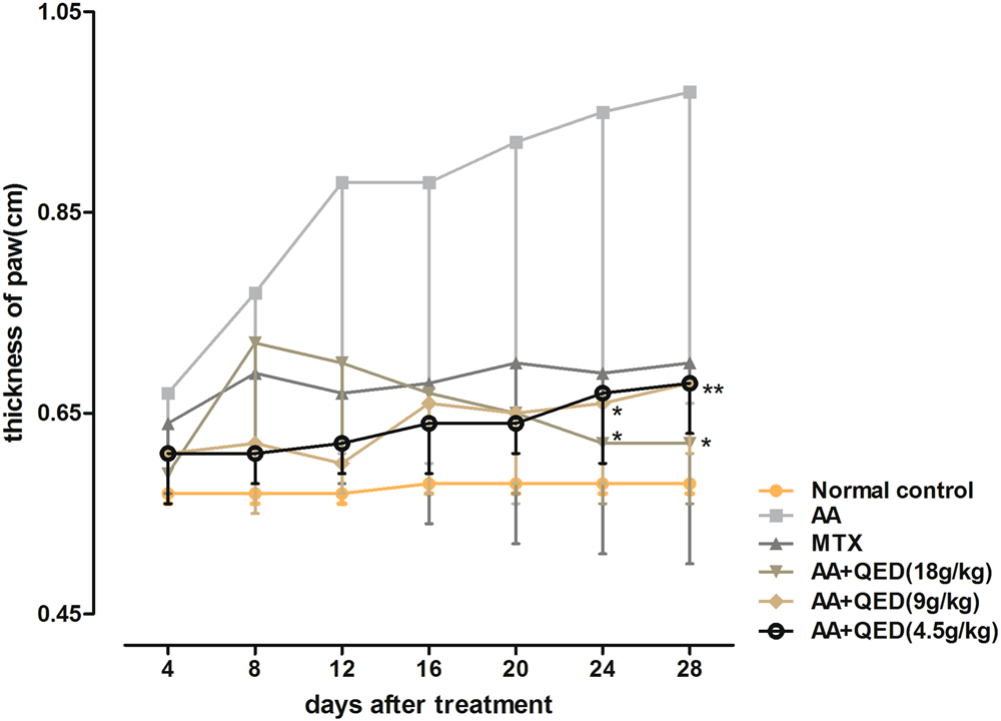
Effects of different QED dosages on the thickness of hind paws. Data are expressed as mean ± S.D. (n = 6 each group).*P < 0.05, **P < 0.01, versus AA group.

After successful induction of arthritis in rats, swelling and thickening of their hind foot palm was observed every four days. Up to 20 days after medication, there was no statistically significant difference (*P* > 0.05) in the foot palm thickness in any treatment group when compared with AA group. In the MTX and high-dose QED groups, foot palm thickness of rats was significantly less on days 24 and 28, respectively after medication. A statistically significant decrease of foot palm thickness in various groups (*P* < 0.05, *P* < 0.01) were observed, which strongly suggested an attenuation of foot swelling, and alleviation of arthritis (Fig. 2).

### High-dose QED ameliorated synovial tissue inflammation in AA rats

**Figure 3:**
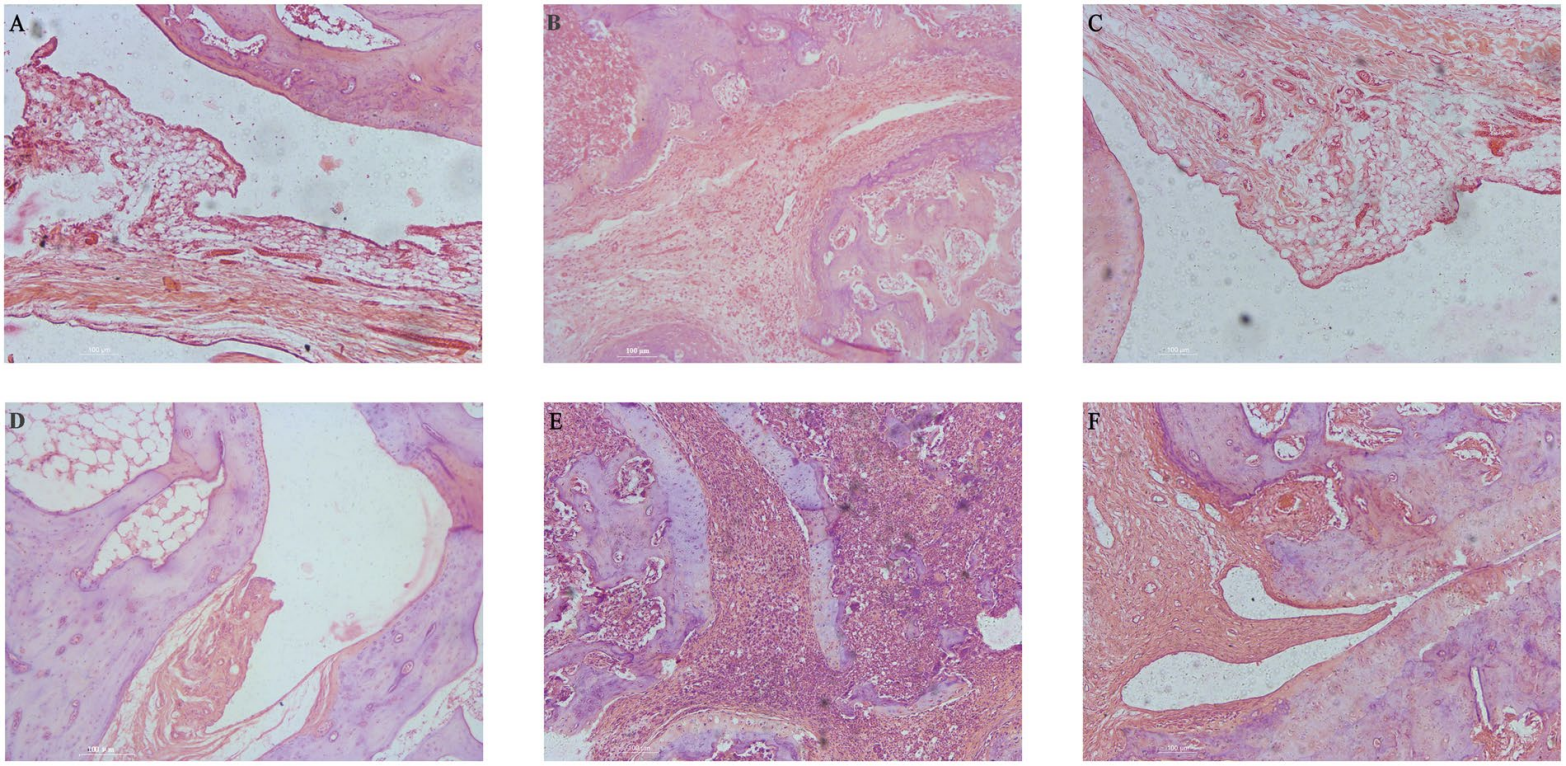
Effects of QED on histological parameters in the talocrural joints of AA rats. Representative histological observation from light microscope of the talocrural joint sections stained with HE for inflammatory cell influx and bone destruction (magnifcation × 100). (**A**) Normal control; (**B**) AA; (**C**) MTX; (**D**) AA + QED (18 g/kg); (**E**) AA + QED (9 g/kg); (**F**) AA + QED (4.5 g/kg).

The normal control group showed smooth articular cartilage tissue and the synovium contained a monolayer of cells without any obvious thickening (A). Rats in the AA group showed thickening of synovial tissue, narrowing of joint space, blood vessel proliferation, and enormous inflammatory cell infiltration, as well as cartilage and bone tissue destruction (B). Rats in the MTX and high-dose QED groups showed thickening of joint synovial tissue, which was less severe when compared with AA group. Moreover, inflammatory cell infiltration was also reduced without any signs of cartilage or bone tissue destruction (C, D). Rats in the moderate- and low-dose QED groups exhibited obvious joint synovial hyperplasia, narrowing of joint space and cartilage destruction (E, F) (Fig. 3).

### High-dose QED inhibited the proliferation of splenocytes in rats

**Figure 4:**
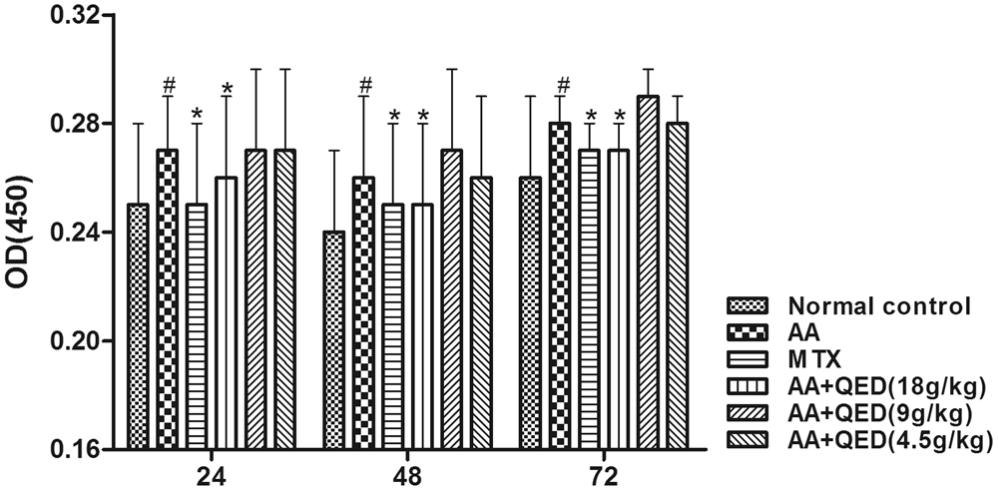
Splenocytes isolated from rats were stimulated with PMA (30 ng/mL) and cultured for 24, 48 and 72 h, respectively. Cell proliferation was measured by the CCK-8 assay. Data are expressed as mean ± S.D (n = 6 each group); ^#^ *P* < 0.05, versus control group; *P < 0.05, versus AA group.

Stimulation with Phorbol myristate acetate (PMA) 30 ng/mL for 24, 48 and 72 h has triggered splenocyte proliferation. Compared with the normal control group, stimulation of rats in the AA group for 24, 48 and 72 h has significantly increased the proliferation of splenocytes (*P* < 0.01). Moreover, compared with the AA group, splenocyte proliferation was obviously inhibited in MTX and high-dose QED groups after stimulation for 24, 48 and 72 h (*P* < 0.01). However, no statistically significant difference was seen between moderate- and low-dose QED groups and AA group (*P* > 0.05; Fig. 4).

### High-dose QED elevated the percentage of Treg cells while decreasing the percentage of Th17 cells in splenocytes

**Figure 5:**
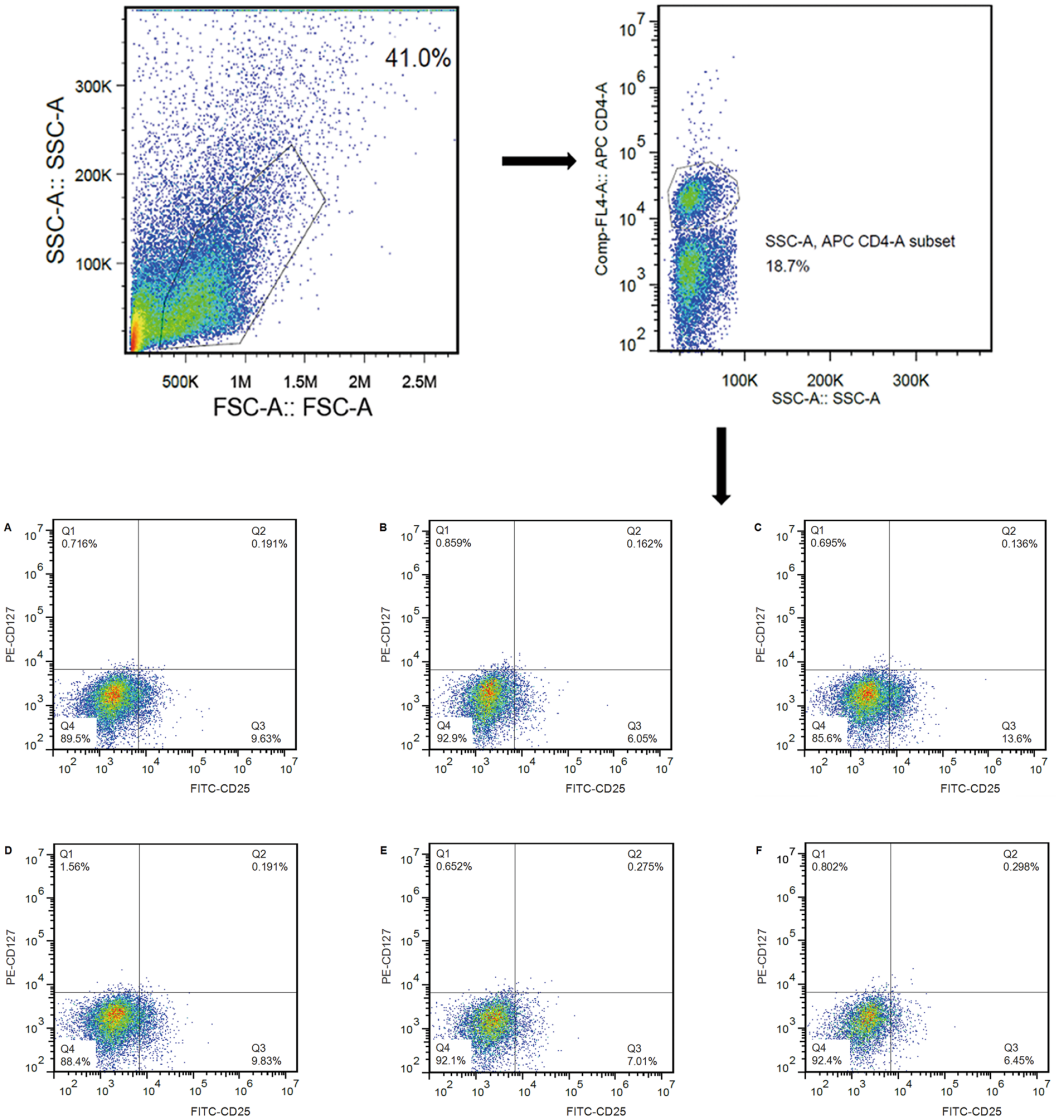
Effects of different QED dosages on the percentage of Treg cells in splenocytes. Isolated splenocytes were quantified and surface stained with various anti-rat antibodies, including APC-conjugated anti-CD4, FITC-conjugated anti-CD25 and PE-conjugated anti-CD127 monoclonal antibodies. Representative graphs showing the percentages of positive-stained CD25^+^CD127^−^ in CD4 + cells analyzed by flow cytometry. (**A**) Normal control; (**B**) AA; (**C**) MTX; (**D**) AA + QED (18 g/kg); (**E**) AA + QED (9 g/kg); (**F**) AA + QED (4.5 g/kg).

**Figure 6:**
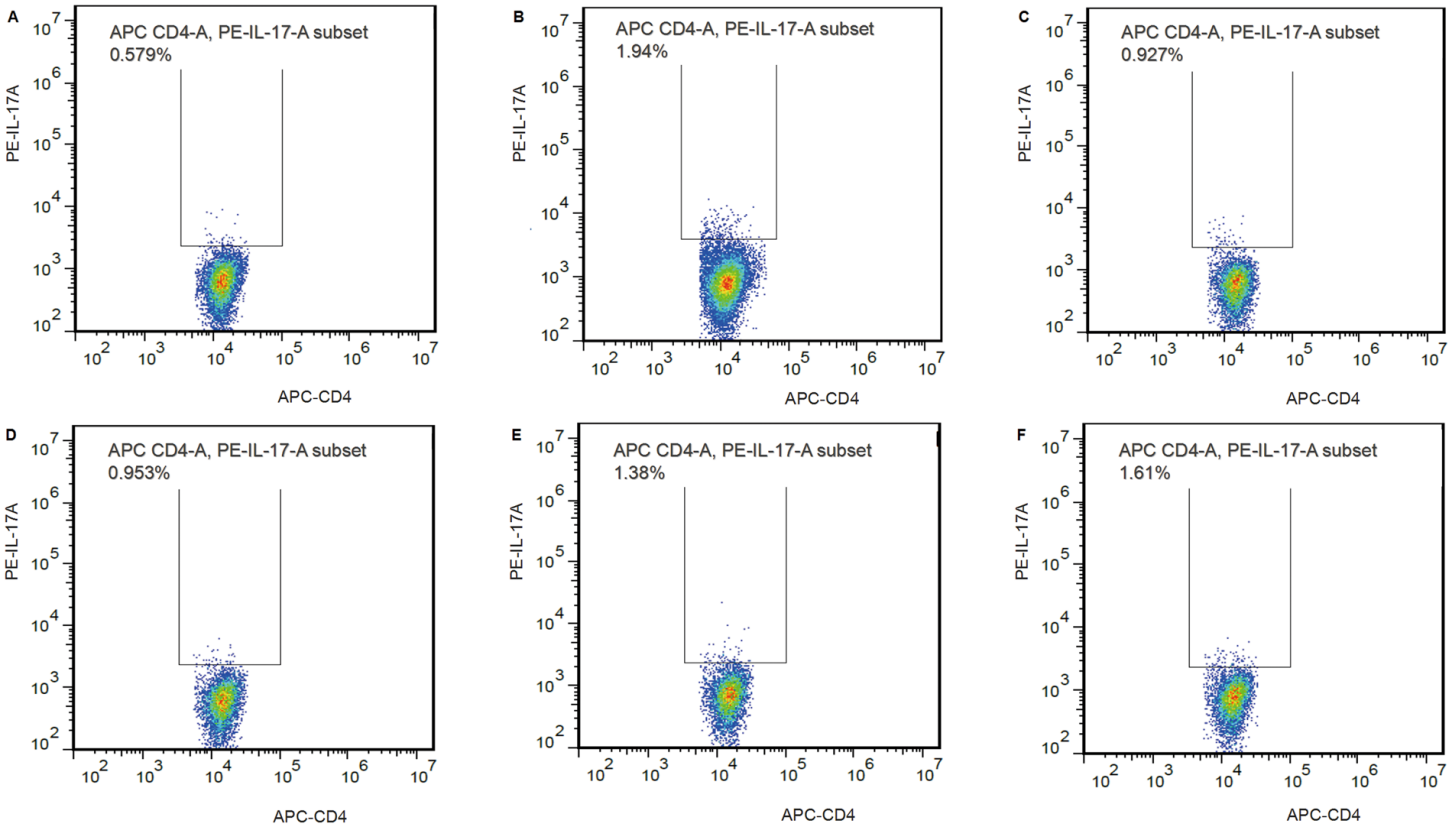
Effects of different QED dosages on the percentage of Th17 cells in splenocytes. Isolated splenocytes were stimulated with PMA (0.05 μg/mL) and ionomycin (1 μg/mL) for 4 h and then BFA (5 μg/mL) for additional 2 h. Cells were stained extracellularly with APC-conjugated anti-CD4, fixed, permeabilized, and labeled intracellularly with PE-conjugated anti-IL-17A. Representative graphs showing the percentages of positive-stained Th17 in CD4 + cells analyzed by flow cytometry. (**A**) Normal control; (**B**) AA; (**C**) MTX; (**D**) AA + QED (18 g/kg); (**E**) AA + QED (9 g/kg); (**F**) AA + QED (4.5 g/kg).

Treg/Th17 cell imbalance has triggered rheumatoid arthritis and lead to severe inflammation. Compared with the normal control group, the percentage of Tregs in the splenocytes of rats in the AA group decreased ((5.75 ± 0.46) %, *P* < 0.01), while the percentage of Th17 increased significantly [(1.84 ± 0.45) %, *P* < 0.01]. The percentage of Treg cells in the high-dose QED group increased significantly [(11.19 ± 2.01) %, *P* < 0.01], while the percentage of Th17 cells declined significantly [(0.98 ± 0.35) %, *P* < 0.05] compared with the AA group. Differences in the percentage of Treg cells and Th17 cells between moderate- and low-dose QED and AA groups showed no statistically significant difference (*P* > 0.05) (Table 1, Figs 5 and 6).

**Table 1:** The proportion of Treg and Th17 cells in splenocytes treated with different QED dosages in rats.

Groups	Treg (%)	*P value*	Th17 (%)	*P value*
Normal control	9.20 ± 0.58		0.67 ± 0.11	
AA	5.75 ± 0.46	*P* = 0.000^##^	1.84 ± 0.45	*P* = 0.004^##^
MTX	11.27 ± 2.29	*P* = 0.000**	0.83 ± 0.22	*P* = 0.010*
AA + QED (18 g/kg)	11.19 ± 2.01	*P* = 0.000**	0.98 ± 0.18	*P* = 0.041*
AA + QED (9 g/kg)	6.61 ± 0.96		1.64 ± 0.78	
AA + QED (4.5 g/kg)	6.58 ± 0.70		1.91 ± 0.45	
Homogeneity test of variance	*P* = 0.056		*P* = 0.062	

Each value represents the mean ± S.D. for six independent experiments. ^#^ *P* < 0.05, ^##^ *P* < 0.01 versus the normal control group. *P < 0.05, **P < 0.01 versus the AA group.

### Effects of different dosages of QED on the serum levels of IL-6, IL-17, TNF-α and TGF-β

**Figure 7:**
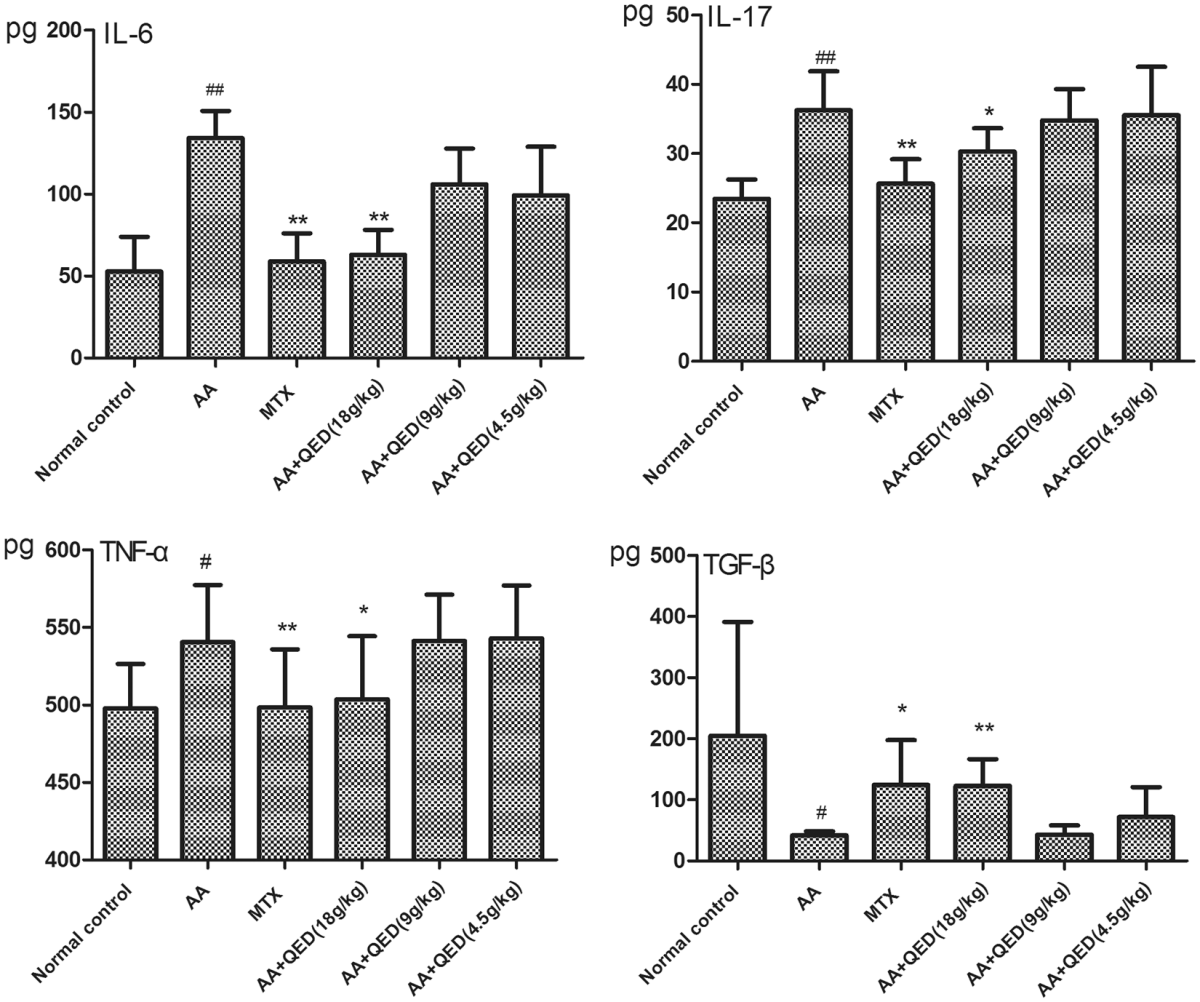
Effects of different dosages of QED on the serum levels of IL-6, IL-17, TNF-α and TGF-β measured by ELISA. Data are expressed as mean ± S.D (n = 6 each group). ^#^ *P* < 0.05, ^##^ *P* < 0.01, compared with the control group; *P < 0.05, **P < 0.01, compared with the AA group.

Serum cytokines were closely related to the degree of RA inflammation and disease activity. IL-6, IL-17 and TNF-α played an important role in the inflammatory response. Our results demonstrated that IL-6, IL-17 and TNF-α levels in rats of the AA group were 134.21 ± 16.58 pg/mL (*P* = 0.000), 36.26 ± 5.62 pg/mL (*P* = 0.001) and 540.01 ± 36.94 pg/mL (*P* = 0.013), respectively, which was higher than in rats in the normal control group. When compared with the AA group, serum cytokine levels were significantly decreased in the MTX and high-dose QED groups: 58.84 ± 17.13 pg/mL, *P* = 0.000; 25.63 ± 3.54 pg/mL, *P* = 0.000; and 498.31 ± 37.50 pg/mL, *P* = 0.006. However, moderate- and low-dose QED did not inhibit the secretion of pro-inflammatory serum cytokines (*P* > 0.05). TGF-β is an important cytokine that inhibits inflammation in the body. Our results showed that compared with the AA group, MTX and high-dose QED groups had a significantly higher levels of TGF-β in rats with arthritis (*P* < 0.05, *P* < 0.01), while moderate- and low-dose QED had no such effect (Fig. 7).

## Discussion

In this study we have successfully developed adjuvant-induced arthritis rats as animal models, which is commonly acknowledged as one of the most widely used models for studying the anti-inflammatory and anti-rheumatic effects of compounds. It is an excellent model to study experimental immunopathology of rheumatoid arthritis as it shares many features with this characteristic clinical condition, such as infiltration of T and B cells, joint synovial hyperplasia, pannus and destruction of articular cartilage or joint fusion^12–14^. Interestingly, the levels of anti-citrullinated peptide (anti-CCP) antibody and pro-inflammatory cytokines (IL-1, TNF-α) were significantly increased. However, the effects on the digestive, reproductive and cutaneous systems were similar to clinical RA, which were characterized by multi-system involvement and chronic changes. This model showed stability and high reproducibility^15,16^. Our experimental results showed that adjuvant administration triggered erythema and ulcer at the injection site initially, followed by erythema of the ear, subcutaneous nodules in the tail, swelling of the toes and the paw, and joint limitation.

Clinically, RA patients during their active phase of disease usually present characteristic symptoms, such as local joint swelling and thermal pain. Clinical and biochemical indices such as ESR, rheumatoid factor (RF) and anti-CCP antibody changes to abnormal levels^1^. Traditional Chinese medicine (TCM) has been widely used to treat RA effectively for thousands of years in China. Our results showed that high-dose QED reduced the arthritis score and relieved joint swelling in animal models. Compared with the AA group, joint synovial hyperplasia of rats treated with a high-dose QED was decreased and showed limited angiogenesis, without any obvious articular cartilage destruction. Furthermore, high-dose QED could inhibit splenic lymphocyte proliferation on stimulation with PMA in rats with AA. On the other hand, moderate- and low-dose QED had no significant therapeutic or anti-proliferation effects. Changes in joint pathology were similar to those in AA rats. Accordingly, the previous results indicated that high-dose QED treatment for adjuvant arthritis showed good therapeutic efficacy, and was associated with inhibition of lymphocyte proliferation.

In recent years, several studies investigated the role of T lymphocyte subpopulations in RA pathogenesis. Th17 cells, by virtue of their production of IL-17 are previously thought to be pro-inflammatory cells playing a major role only in autoimmunity, are now shown to facilitate host immune response against infections with extracellular bacteria and fungi. The Treg cells were also shown to mediate immune tolerance and maintain lymphocyte homeostasis. Pathogenic Th17 cells are reported to mediate pannus growth, osteoclastogenesis, and synovial neoangiogenesis, which are the key players in the development of the disease. On the other hand, Treg cells are T cell subsets that suppress auto-reactive lymphocytes^17^. Most studies reported a diminished number of circulating Treg cells and a higher level of circulating Th17 cells in RA compared with healthy subjects, suggesting that imbalance between Th17 and Treg cells is a key event in RA pathogenesis^18,19^.

Treg cells are considered as the critical regulators of immune tolerance, and most of them are defined by the expression of CD4, CD25, and a transcription factor, FoxP3^20^. However, these markers do not facilitate the complete characterization of this specialized T cell subset^21^. It was found that the IL-7 receptor (CD127) is down regulated on a subset of CD4^+^ T cells in peripheral blood. The majority of these cells are FoxP3^+^, including those that express low levels or no CD25. A combination of CD4, CD25, and CD127 receptors facilitated the purification of Treg cell population in significantly higher numbers, compared with previous attempts based on other cell surface markers^22,23^. Thus, the low expression of CD127 combined with a high expression of CD25 facilitated the differentiation and purification of Treg cells in CD4^+^ CD25^+^ T cells. Moreover, the function of Treg cells expressing CD4 ^+^ CD25^high^ CD127^low^ was highly inhibitory^24,25^. Most studies reported that a reduced number of circulating Treg cells and a higher levels of circulating Th17 cells in RA compared with healthy subjects, strongly suggested an imbalance in Th17/Treg ratio, which may be a crucial event in RA pathogenesis^18,19^. It was shown that, the percentage of splenic Treg cells in AA group was significantly decreased, while that of Th17 cells was apparently elevated compared with normal rat. The percentage of splenic Treg cells was significantly increased with high-dose QED and methotrexate treatment for four weeks. However, the Th17 cells were significantly decreased, indicating that high-dose QED and methotrexate regulated Treg/Th17 ratio. Granuloctye-macrophage colony stimulating factor (GM-CSF) promotes Treg expansion and reported to play a major role in suppressing autoimmune disease. In terms of Treg and Th17 mediated autoimmune response, the GM-CSF also could play an important role in many diseases, such as Crohn’s Disease, diabetes etc^26–28^. According to our study, the major effect of QED down-regulated the Th17 ratio while up-regulating the Treg ratio, however, whether QED is associated with GM-CSF requires more in-depth exploration. It was shown that, the treatment of type II collagen-sensitive arthritis mice was closely related to peripheral immune tolerance, however the exact role of this interaction in the AA rat is unclear, which also needs further exploration^29,30^.

Mounting evidence in animal and human studies supports that pro-inflammatory cytokine IL-17 is specific for Th17 cells, which lead to chronic tissue inflammation and organ fibrosis, and is considered as a key factor in RA pathogenesis^31^. Multiple regression analysis revealed that the increased expression of IL-17, tumor necrosis factor (TNF)-α and IL-1 mRNA in the synovial tissue of RA were predictors of joint destruction^32^. Differentiation of Th17 cells was closely related to transforming growth factor (TGF)-β and IL-6 levels, while TGF-β was essential for Treg cell differentiation^33,34^. Compared with normal control group, the levels of IL-6, IL-17, and TNF-α in the peripheral blood of AA group were higher, whereas TGF-β level was inadequate. TGF-β levels were significantly increased after exposure to high-dose QED and methotrexate, and the secretion of IL-6, IL-17, and TNF-α was significantly reduced. High-dose QED treatment significantly decreased the serum levels of IL-6, IL-17 and TNF-α, while the TGF-β levels were apparently elevated compared with AA group, suggesting that high-dose QED regulated the network of related cytokines and restored the balance between Treg and Th17.

In summary, high dosage of QED was effective against adjuvant arthritis and in regulating the levels of serum cytokines. The underlying mechanism might be mediated via restoration of CD4^+^ T lymphocyte balance involving Treg/Th17 ratio. Interestingly, moderate- and low-dose QED showed no apparent therapeutic effect when compared with the AA group, which indicated that the high QED dose had a significant therapeutic effect. This study found that the effect of QED and positive control MTX had no statistical significance, suggesting that the Chinese prescription was effective and should be recommended in treating RA. However, the side effects in this study were not completely elucidated. Further studies are needed to determine the safety and pharmacokinetics of QED before approving its wide clinical application.

## Materials and Methods

### Animal study

Six- to eight-week-old male specific pathogen-free Sprague-Dawley (SD) rats, each weighting 200 ± 20 g (Permit Number: SCXK (JUN) 2012–0009) were obtained from the Experimental Animal Center of the Third Military Medical University (Chongqing, China). Animal protocols were approved by the Animal Care and Use Committee of the Southwest Hospital, Third Military Medical University, China, and were in accordance with “Principles for Use of Animals” and “Guide for the Care and Use of Laboratory Animals” of the U.S. National Institutes of Health. All the animals were maintained in cages at 20 ± 2 °C with free access to pelleted food and water and were maintained under a 12 h light/12 h dark cycle. This study complied with current ethical regulations involving animal research at our institute. All the animals used in the study received humane care.

### Reagents and drugs

Phorbol myristate acetate (PMA), dimethyl sulfoxide (DMSO) and methotrexate (MTX) were obtained from Sigma (St Louis, MO). CCK-8 cell proliferation and cytotoxicity assay kits were supplied by KeyGEN (Nanjing, China). Cell stimulation and protein inhibition cocktails were purchased from eBioscience Biotech (CA, USA). APC-conjugated anti-rat CD4 and FITC-conjugated anti-rat CD25 monoclonal antibodies were obtained from BioLegend Biotech (CA, USA). PE-conjugated anti-rat CD127 and IL-17A monoclonal antibodies were purchased from R&D Biotech (MN, USA). Tumor necrosis factor-alpha (TNF-α), transforming growth factor-beta (TGF-β), interleukin-6 (IL-6) and IL-17 ELISA kits were purchased from Elabscience Biotech (GA, USA).

### Herbal ingredients

The medicinal ingredients of QED recipe were purchased from Sanjiu Medical and Pharmaceutical Groups (Shenzheng, China), and carefully authenticated by colleagues in Department of Pharmacology of Third military medical university (Chongqing, China). The mixture was decocted twice by refluxing with water (1:10 and then 1:5 w/v) for 30 min, the drug suspension was lyophilized, and subjected to High Performance Liquid Chromatography fingerprinting^35^. Drug powder was dissolved in normal saline to a final concentration of 1.8 g/mL (equivalent to the dry weight of the raw materials).

### Induction and evaluation of adjuvant arthritis (AA) in rats

AA was induced by the same procedures described in previous studies^13^. Arthritis was induced in SD rats via subcutaneous injection at the tail base with 200 μL of heat-killed *Mycobacterium tuberculosis* H37R (Mtb) (Difco, Detroit, MC. Gift from Professor Li Tong) at a dose of 1.0 mg/rat in mineral oil. After the immunization, rats were examined once daily and graded according to the signs observed since the onset of arthritis (on day 14). The four limbs of the rats were monitored every 24 h by visually assessing inflammation or swelling. The arthritis score was determined as described: 0 = normal with no erythema and swelling; 1 = swelling and erythema of the digit, 2 = mild swelling and erythema of the limb, 3 = moderate swelling extending from the ankle to the metatarsophalangeal or metacarpophalangeal joints, and 4 = severe swelling extending from the ankle to the digits, resulting in ankylosis and loss of joint movement. The arthritis score of each rat was the sum of the scores involving the four paws, with a maximal score of 16 per rat^36^. The arthritis score and the thickness of the inflamed hind paws were determined every day from day 1 to day 28, after the animal model was established.

### Animal grouping and drug treatment

Thirty-six rats were randomly divided into 6 groups as follows: normal control group (oral intake of normal saline); AA group (normal saline); AA + methotrexate group (MTX, 0.4 mg/kg of body weight per week); AA + high-, moderate- and low-dose QED groups (daily dose: 18, 9 and 4.5 g/kg of body weight, respectively). From the first day of joint swelling, the rats were administered with the corresponding agents through oral gavage. The doses in the treatment group were converted from human doses (Chinese Pharmacopeia, 2010) based on body surface area.

### Hematoxylin-eosin (HE) staining of synovial tissue from talocrural joints

Rats in all the groups were sacrificed after 4 weeks of treatment by cervical vertebra dislocation after anesthetizing with 4% pentobarbital solution. Right hind limbs were fixed in 4% neutral formalin for one week and decalcified in 0.5 mmol/L ethylene diamine tetraacetic acid (EDTA) and 0.5% paraformaldehyde for 20 days. Decalcified samples were dehydrated with alcohol, embedded in paraffin and sectioned into 10-μm-thick specimens. Talocrural joint sections were stained with hematoxylin and eosin and mounted on glass slides for histological analysis under the light microscope.

### Splenocyte proliferation

Rat spleens were removed under aseptic conditions, and the Red blood cells in the suspension were removed by treatment with 0.16 M Tris-NH_4_Cl solution prior to preparation of single-cell suspension of splenocytes. Splenocytes (2 × 10^6^ cells/mL) were seeded in 96-well plates at 2 × 10^5^ cells/well in an RPMI 1640 medium supplemented with 50 IU/mL penicillin, 60 mg/mL streptomycin and 5% inactivated fetal bovine serum (all from Invitrogen). After incubation for 12 h at 37 °C in humidified air containing 5% CO_2_, the cells were stimulated with PMA (30 ng/mL) for 24, 48 and 72 h at 37 °C, respectively. After addition of 10 μL of CCK-8 solution to each well, plates were incubated for 4 h at 37 °C. The absorbance of each well at 450 nm was read using a spectrophotometer (Thermo, USA). Proliferation assays were conducted using cultures and was repeated in triplicate^37^.

### Flow cytometric analysis (FACS) of Treg and Th17 cells from splenocytes

Splenocytes (2 × 10^6^ cells/mL) were prepared from the spleens of all rats as described previously. The cells were quantified and surface stained with various anti-rat antibodies conjugated with different fluorescent markers, including APC-conjugated anti-CD4, FITC-conjugated anti-CD25 and PE-conjugated anti-CD127 monoclonal antibodies. Acquisition and analysis were conducted using BD FACScalibur and CellQuest Pro software (BD Bioscience) after gating on live cells based on scatter characteristics according to the manufacturer’s protocol^38^.

Splenocytes (2 × 10^6^ cells/mL) were stimulated with PMA (0.05 μg/mL) and ionomycin (1 μg/mL) for 4 h, followed by brefeldin A (BFA, 5 μg/mL) for 2 h. Cells were stained extracellularly with APC-conjugated anti-CD4, fixed, permeabilized, and labeled intracellularly with PE-conjugated anti-IL-17A^39^. The proportion of stained cells was measured using FACS instruments. Data were analyzed using FlowJo software (Tristar).

### Enzyme-linked immunosorbent assay (ELISA) of serum cytokines

Before sacrificing, serum samples were separated from collected heart blood from all rats in every group. The levels of interleukin (IL)-6, IL-17, tumor necrosis factor (TNF)-α and transforming growth factor (TGF)-β in the serum were determined by ELISA kits according to the specifications, in triplicate for each sample.

### Statistical analysis

All data with a normal distribution were expressed as mean ± standard deviation and analyzed using SPSS13.0 statistical software. Statistical significance was determined by one-way analysis of variance (ANOVA). Data with equal variances were analyzed with ANOVA followed by LSD test. ANOVA followed by Dunnett’s T3 test was used for analyzing data that were not assuming equal variances. A probability of less than 0.05 was considered to be statistically significant.
